# Supplementary material for: Spectroscape enables real-time query and visualization of a spectral archive in proteomics
Source: Nat Commun. 2023 Oct 7;14:6267. doi: 10.1038/s41467-023-42006-x (PMC10560257; doi:10.1038/s41467-023-42006-x)
Supplement: Supplementary file 1 — Supplementary information [file 41467_2023_42006_MOESM1_ESM.pdf]

Spectroscape Enables Real-time Query and Visualization of a Spectral Archive in Proteomics

Long Wu[1,2], Ayman Hoque[1], Henry Lam[1,*]

1 Department of Chemical and Biological Engineering, The Hong Kong University of Science and Technology

2 Department of Electrical and Computer Engineering, The Hong Kong University of Science and Technology

Supplementary Information

* Corresponding author

Henry Lam

Department of Chemical and Biological Engineering

The Hong Kong University of Science and Technology

Clear Water Bay

Hong Kong Special Administrative Region

China

Email: kehlam@ust.hk

Table of Content

Supplementary Figures Page

Supp. Figure 1 | Optimization of the hyper-parameters *nprobes* and *nindices* ……………………..3

Supp. Figure 2 | More examples of visualized spectrum clusters by Spectroscape ………………. 4

Supp. Figure 3 | The default interface of Spectroscape UI ………………………………..…… 5

Supp. Figure 4 | The star connectivity with concentric circle plot of Spectroscape UI…………… 6

Supp. Figure 5 | The butterfly spectrum-to-spectrum match viewer of Spectroscape UI………… 7

Supp. Figure 6 | The peak list search function of Spectroscape UI ….…………………………… 8

Supp. Figure 7 | The node table of Spectroscape UI……………… ……………………………… 9

Supplementary Tables

Supp. Table 1 | Speed comparison of Spectroscape and ANN-Solo ……………………………… 10

Supp. Table 2 | Search result comparison between Spectroscape and ANN-Solo ..……………… 11

Supp. Table 3 | Re-annotation of unidentified and misidentified spectra by spectrum clustering… 12

Supplementary Notes

Supp. Note 1 | Open modification spectral library search and comparison with ANN-Solo …… 13

Supp. Note 2 | Offline clustering and re-annotation of unidentified and misidentified spectra … 14

Supplementary Methods

Supp. Method 1 | The FAISS IVF-PQ algorithm ………………………………………………… 15

Supp. Method 2 | Optimization of the FAISS IVF-PQ algorithm for spectral archives …………. 18

Supp. Method 3 | Comparison with GLEAMS in terms of ANN retrieval ……….……………… 18

Supp. Method 4 | True dot product calculations …………………………………………………. 20

Supp. Method 5 | Visualization and force-directed graph drawing ……………………………… 21

Supplementary Figures

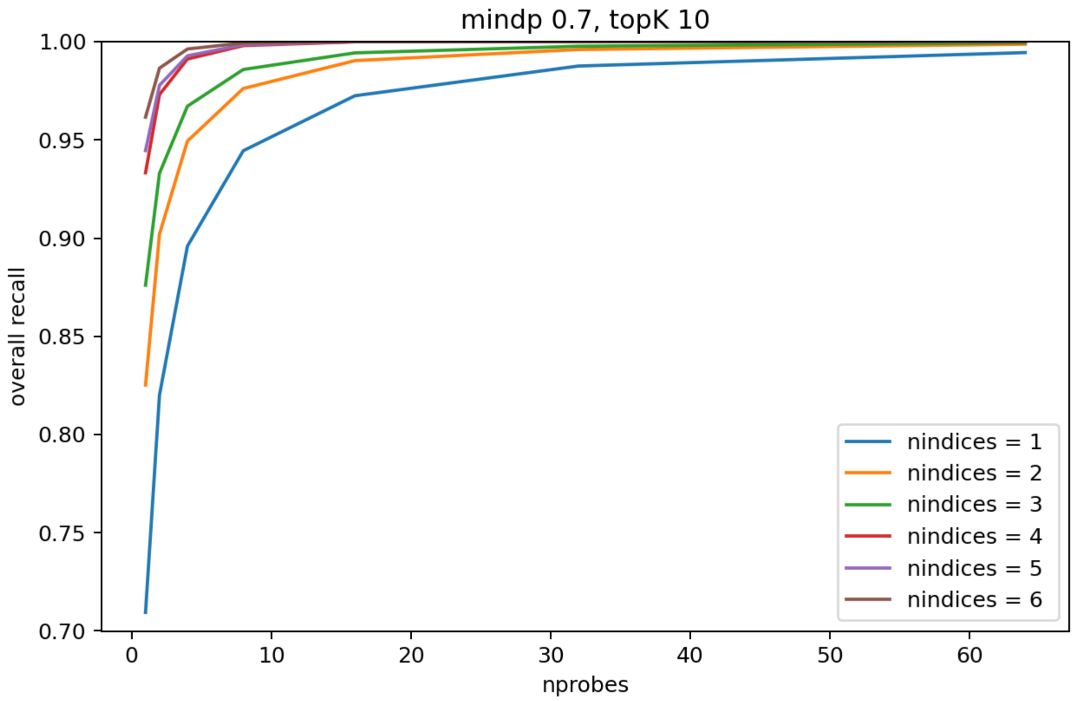

Supplementary Figure 1 | Optimization of the hyper-parameters *nprobes* and *nindices*. The overall recall of a randomly selected set of 10,000 queries was determined for each parameter setting. As shown, the overall recall improves when *nprobes* and *nindices* increase, but the improvement diminishes quickly beyond *nprobes* = 8 and *nindices* = 2. To strike a balance between recall and efficiency, the hyper-parameter setting of *nprobes* = 8 and *nindices* = 2 was chosen for this study. The overall recall is defined as:

$$R_{overall} = \frac{\sum_{i=1}^{10000} |A(\boldsymbol{q_i}) \cap T(\boldsymbol{q_i}, N, t)|}{\sum_{i=1}^{10000} |T(\boldsymbol{q_i}, N, t)|}$$

where $A(\boldsymbol{q_i})$ is the set of retrieved ANNs of the *i*-th query, and $T(\boldsymbol{q_i}, N, t)$ is the set of retained TNNs of the *i*-th query. The minimum dot product threshold to be retained as TNN, t, is set to be 0.7, and the maximum number of TNNs retained for each query, N, is set to be 10.

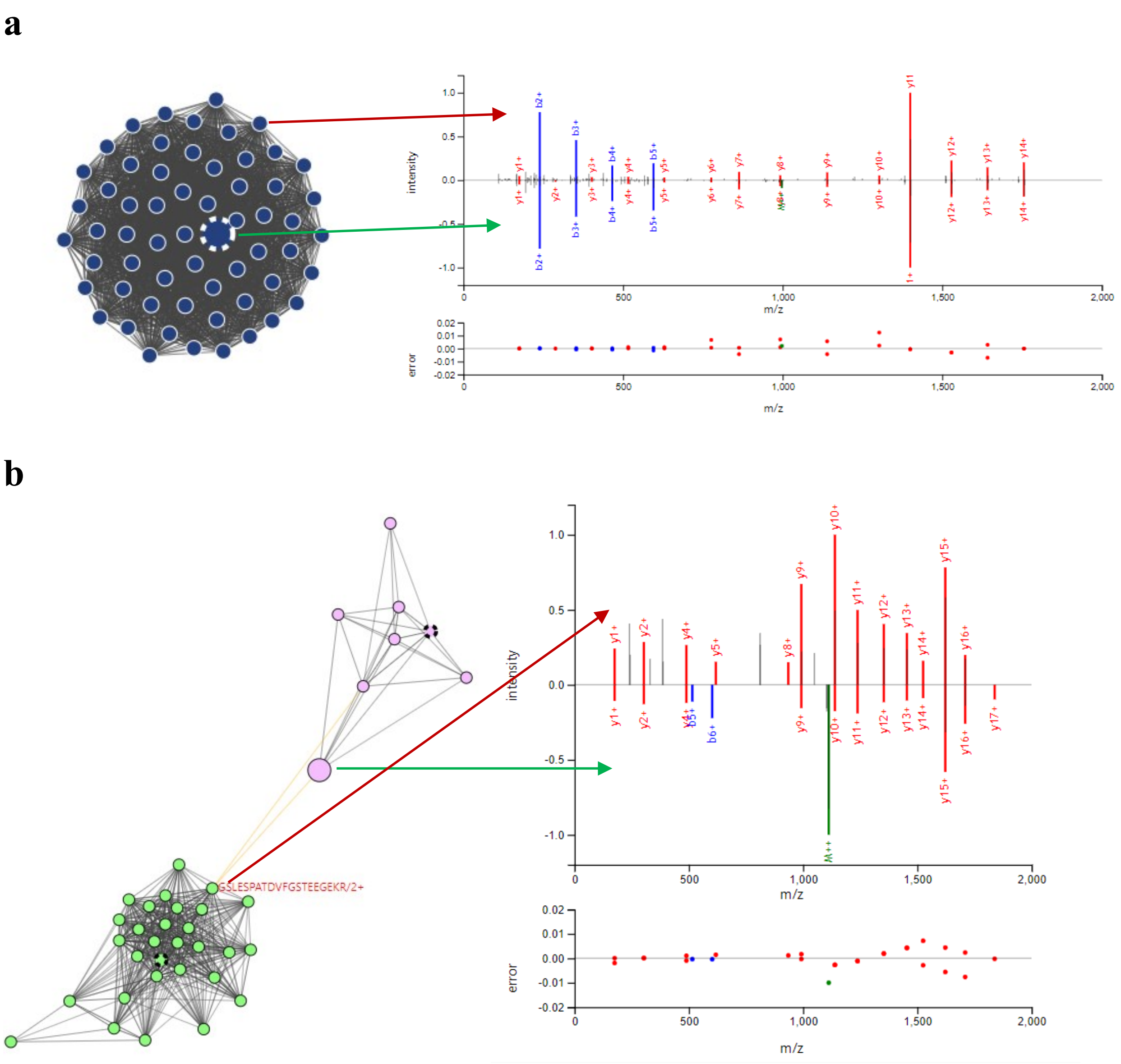

Supplementary Figure 2 | More examples of visualized spectrum clusters by Spectroscape. (a) The consensus library spectrum is near the center of a cluster, shown as the node with dashed outline. This represents the ideal scenario where the replicate spectra are all highly similar to each other, and the averaging scheme to produce the consensus spectrum works as intended. On the right, a butterfly plot showing the spectrum-spectrum match between the library spectrum (down) and one of the replicates (up, its node indicated by red arrow). As shown, even for a neighbor at the edge of the cluster, the spectra are extremely similar. (b) Two clusters colored by pink and green are connected with orange edges, which indicate a precursor mass difference. The spectra in pink and green are identified to peptide KGSLESPATDVFGSTEEGEKR/2, and GSLESPATDVFGSTEEGEKR/2, respectively, the leading K being the only difference and accounting for the precursor mass difference. The butterfly plot on the right shows that the two spectra share many y ions but no b-ions due to the difference at the N-terminus.

Supplementary Figure 3-7 | Screenshots showing the features of Spectroscape. Detailed descriptions are below each screenshot.

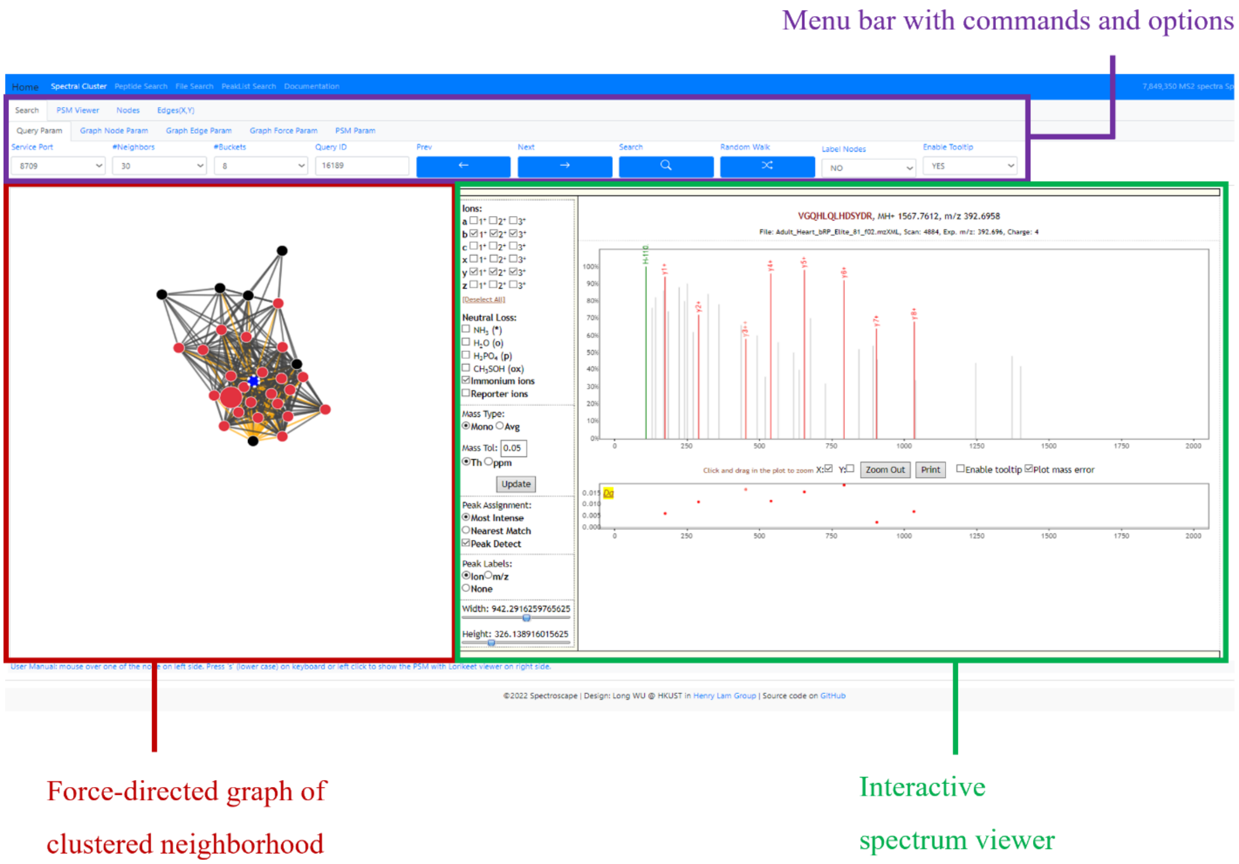

Supplementary Figure 3 | The default interface. The left pane (red box) shows a force-directed graph depicting the clustered "neighborhood" of the query spectrum. The default setting shows the 20 "true" nearest neighbors (small circles). The interactive Lorikeet viewer (green box) on the right shows the 50-peak rank-transformed spectrum of the query (annotated with its putative identification loaded into the archive). A menu bar with many commands and options for the user to execute new queries, explore the archive and customize Spectroscape is on top (purple box).

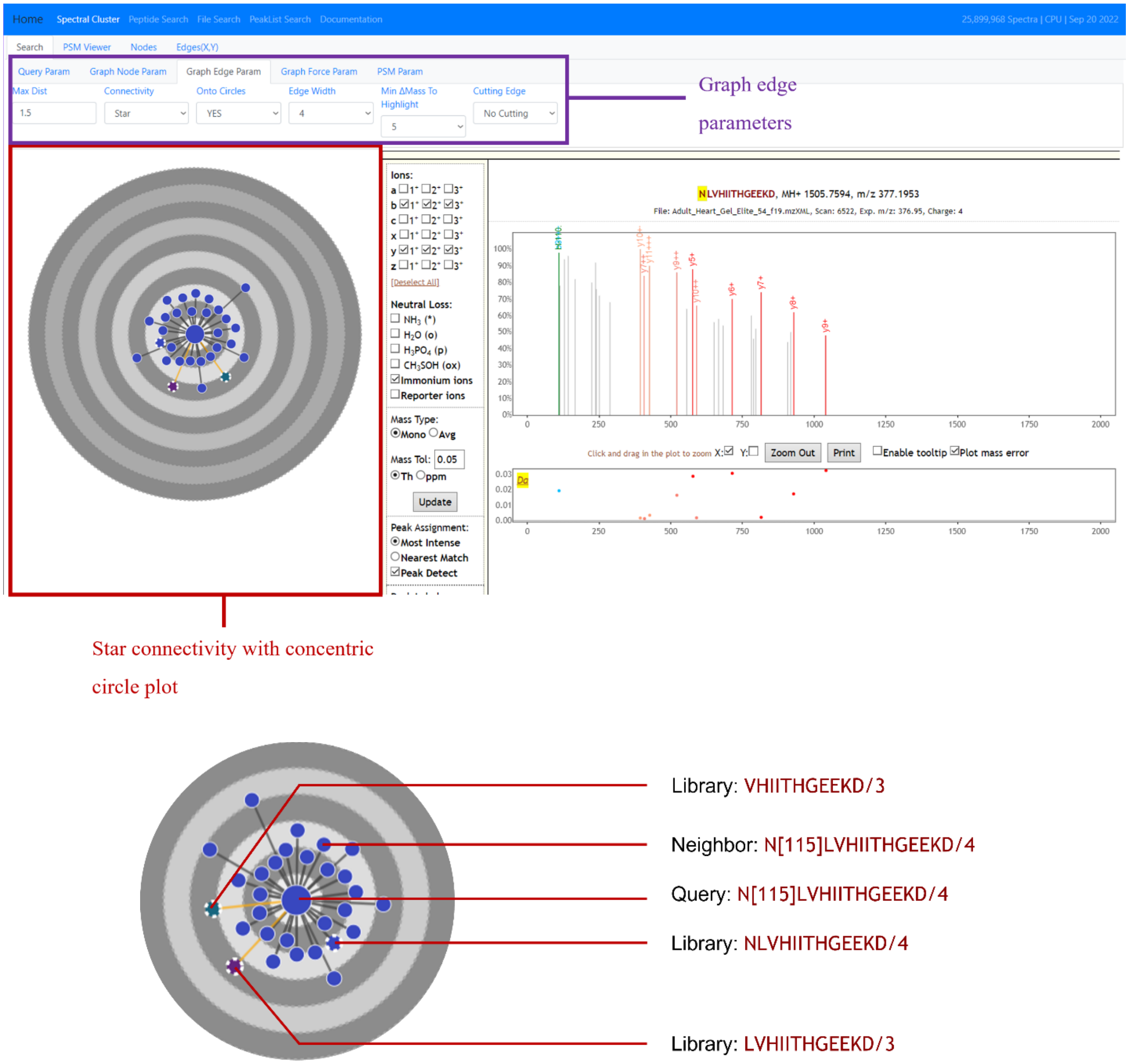

Supplementary Figure 4 | The star connectivity with concentric circle plot. The user can use this visualization feature by selecting “Star” in the “Connectivity” menu in the graph edge parameters (purple box). The star visualization shows only nodes connected to the query node as opposed to all edges between "neighbor" nodes. The concentric circle visualization places each neighboring node into an "orbit" divided according to a dot-product threshold. The star connectivity plot is better for showing the neighbors in the order of similarity. The zoomed-in picture below shows that the query (identified to N[115]LVHIITHGEEKD/4 where N[115] is deamidated asparagine) has neighbors of slightly different sequences, including the library spectrum of unmodified NLVHIITHGEEKD/4 and the library spectra of the N-terminal truncated peptides LVHIITHGEEKD/3 and VHIITHGEEKD/3 of different charge states further away. The orange edges indicate a difference in precursor mass between nodes. The mass difference of deamidation

(+1 Da), on the other hand, is within the user-defined tolerance for precursor mass difference(5 Da as shown above as parameter “minimum Δmass to highlight”), is therefore shown as black.

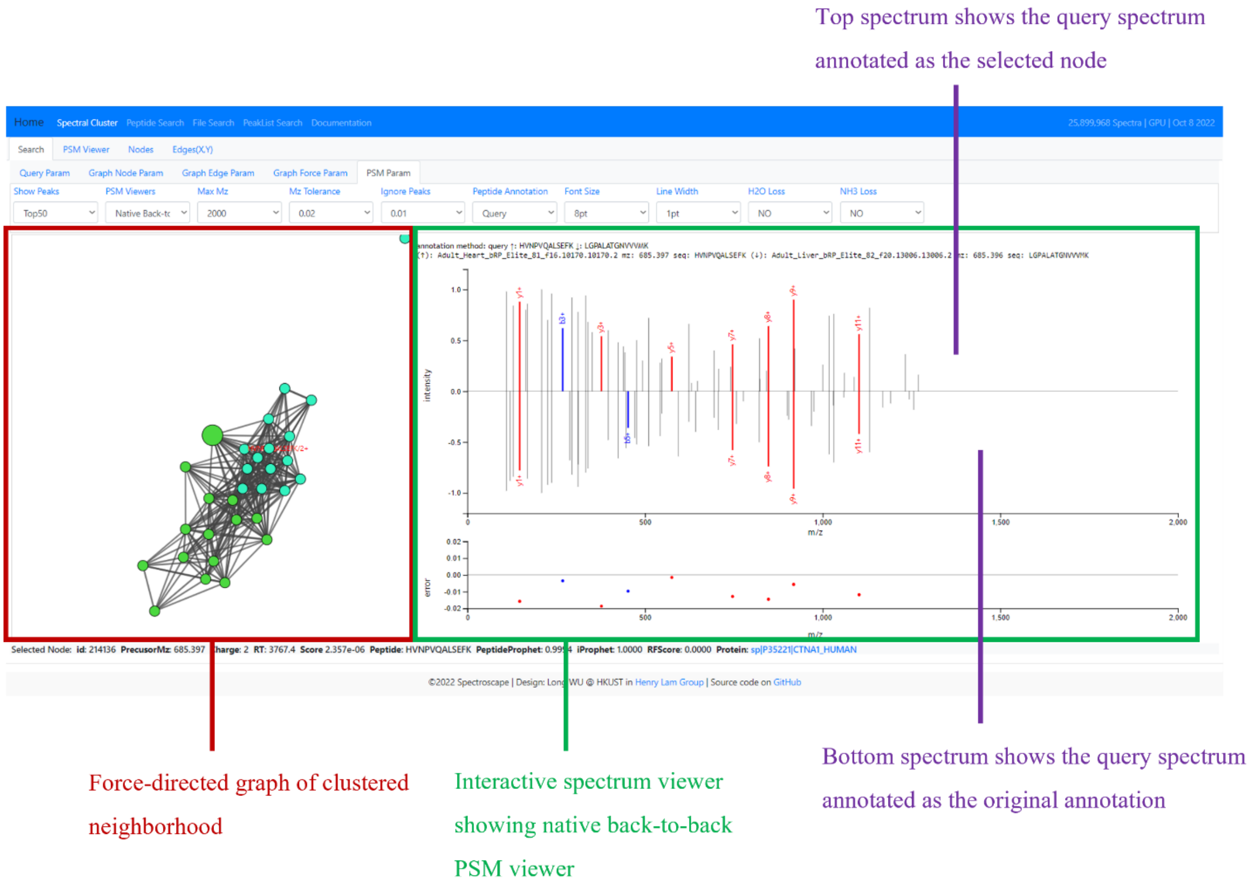

Supplementary Figure 5 | The “butterfly” spectrum-to-spectrum match viewer. Two spectra can be shown “head-to-tail” for easier comparison, and each can be re-annotated with respect to the identification of the other node. The bottom spectrum is of the query node, which is identified by the search engine as LGPALATGNVVVMK/2. The top spectrum is that of the query spectrum annotated as the selected query node which was identified as HVNPVQALSEFK/2. This feature is helpful for correcting the identification of a query node based on its neighbors.

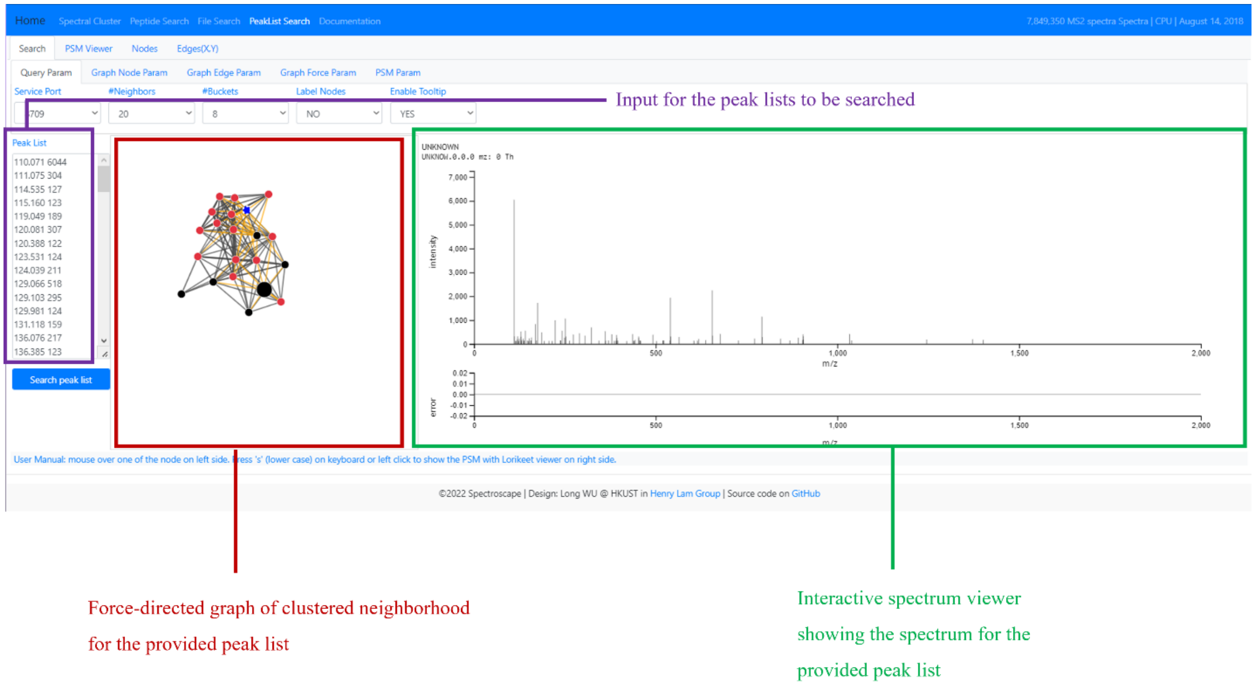

Supplementary Figure 6 | The peak list search function. The left pane (purple box) is an input box for the user to provide a list of peaks to be searched against the archive. Nearest neighbors closest to this provided peak list are retrieved and clustered by Spectroscape (red box). On the right, the provided peak list can be visualized as a spectrum (green box).

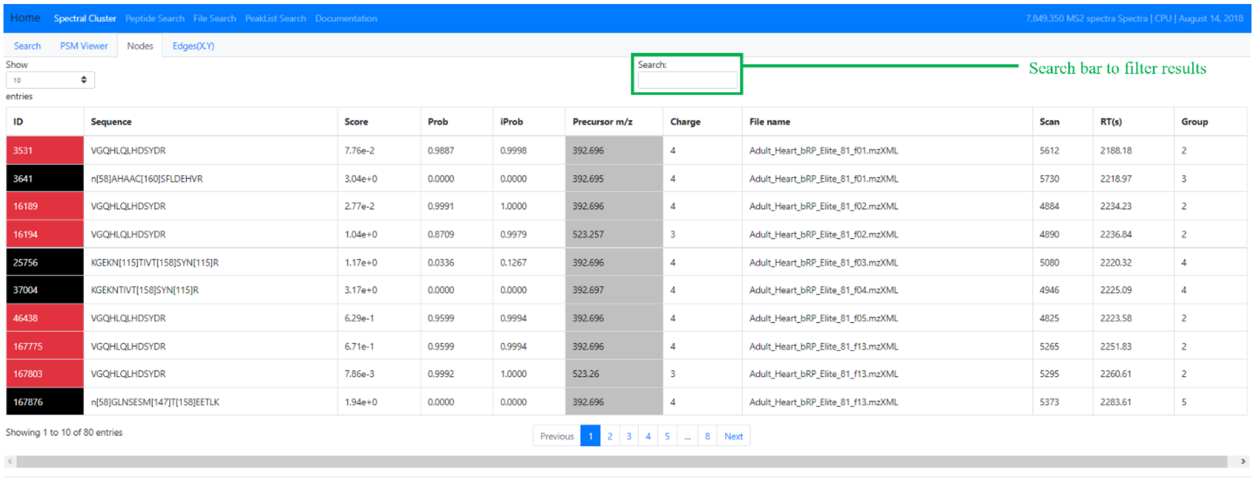

ID	Sequence	Score	Prob	iProb	Precursor m/z	Charge	File name	Scan	RT(s)	Group
3531	VGQHLQLHDSYDR	7.76e-2	0.9887	0.9998	392.696	4	Adult_Heart_bRP_Elite_81_f01.mzXML	5612	2188.18	2
3641	n[58]AHAAC[160]SFLDEHVR	3.04e+0	0.0000	0.0000	392.695	4	Adult_Heart_bRP_Elite_81_f01.mzXML	5730	2218.97	3
16189	VGQHLQLHDSYDR	2.77e-2	0.9991	1.0000	392.696	4	Adult_Heart_bRP_Elite_81_f02.mzXML	4884	2234.23	2
16194	VGQHLQLHDSYDR	1.04e+0	0.8709	0.9979	523.257	3	Adult_Heart_bRP_Elite_81_f02.mzXML	4890	2236.84	2
25756	KGEKN[115]TIVT[158]SYN[115]R	1.17e+0	0.0336	0.1267	392.696	4	Adult_Heart_bRP_Elite_81_f03.mzXML	5080	2220.32	4
37004	KGEKNTIVT[158]SYN[115]R	3.17e+0	0.0000	0.0000	392.697	4	Adult_Heart_bRP_Elite_81_f04.mzXML	4946	2225.09	4
46438	VGQHLQLHDSYDR	6.29e-1	0.9599	0.9994	392.696	4	Adult_Heart_bRP_Elite_81_f05.mzXML	4825	2223.58	2
167775	VGQHLQLHDSYDR	6.71e-1	0.9599	0.9994	392.696	4	Adult_Heart_bRP_Elite_81_f13.mzXML	5265	2251.83	2
167803	VGQHLQLHDSYDR	7.86e-3	0.9992	1.0000	523.26	3	Adult_Heart_bRP_Elite_81_f13.mzXML	5295	2260.61	2
167876	n[58]GLNSESM[147]T[158]EETLK	1.94e+0	0.0000	0.0000	392.696	4	Adult_Heart_bRP_Elite_81_f13.mzXML	5373	2283.61	5

Supplementary Figure 7 | The node table. This feature shows all the neighbors within a certain proximity. The number of neighbors parameter in the Search tab determines the number of nodes shown in the table. By double clicking a column, the table can be sorted according to that column, allowing us to quickly group nodes according to attributes such as Sequence, Probability, and File name. The search bar can also be used to filter the table (green box). Clicking on a cell in the "ID" column will initiate a search using that spectrum as a query.

Supplementary Tables

a. Search time comparison

Dataset	Number of Spectra	ANN-Solo	Spectroscape
**Adult_Heart_bRP_Elite_39_f24**	14498	376 s	117 s
**Adult_Heart_bRP_Elite_39_f08**	15322	357 s	125 s

b. Indexing time comparison

	ANN-Solo	Spectroscape
**Index Time**	612 s	3794 s

Supplementary Table 1 | Speed comparison of Spectroscape and ANN-Solo. (a) Comparison of search time for two files in the PXD000561 dataset. (b) Comparison of indexing time of the NIST Human HCD library (~1.8 million spectra, including decoys).

a. Agreement with ANN-Solo SSMs filtered at 1% FDR

Dataset	ANN-Solo SSMs at 1% FDR	Number of SSMs agreed by Spectroscape	Percentage
**Adult_Heart_bRP_Elite_39_f24**	5339	3710 (3949)	69.5 (74.0)
**Adult_Heart_bRP_Elite_39_f08**	6211	4434 (4782)	71.4 (77.0)

b. Agreement with ANN-Solo SSMs filtered at 0.1% FDR

Dataset	ANN-Solo SSMs at 0.1% FDR	Number of SSMs agreed by Spectroscape	Percentage
**Adult_Heart_bRP_Elite_39_f24**	4919	3629 (3851)	73.8 (78.3)
**Adult_Heart_bRP_Elite_39_f08**	5990	4473 (4813)	74.6 (80.4)

Supplementary Table 2 | Search result comparison between Spectroscape and ANN-Solo. (a) Agreement of Spectroscape's top-ranked SSMs with the SSMs filtered at 1% FDR by ANN-Solo. (b) Agreement of Spectroscape's top-ranked SSMs with the SSMs filtered at 0.1% FDR by ANN-Solo. The number in parentheses are the number of agreed SSMs if we consider top-10 SSMs found by Spectroscape.

	Total number
Identified spectra (identified by MSFragger at 1% FDR)	10,613,412
Unidentified spectra	15,568,853
Unidentified spectra belonging to a cluster	9,512,919
Unidentified spectra belonging to a cluster with at least one identified member	4,005,332
Unidentified spectra belonging to a cluster with a majority identification **(re-annotated)**	589,162
Mis-identified spectra belonging to a cluster with a different majority identification **(re-annotated)**	110,374
All re-annotated spectra	699,536

Supplementary Table 3 | Re-annotation of unidentified and misidentified spectra by spectrum clustering, in a spectral archive of ~26 million spectra in the PXD000561 dataset

Supplementary Notes

Supplementary Note 1. Open modification spectral library search and comparison with ANN-Solo

Spectroscape can be used to perform a conventional library search, with the inherent ability to consider unexpected modification since it does not use the precursor m/z values to select candidates. To demonstrate this potential application, we compared the performance of Spectroscape and ANN-Solo, a open modification spectral library search engine.

ANN-Solo is a software package that utilizes approximate nearest neighbor (ANN) search algorithms to perform open modifications search against spectral libraries. Like Spectroscape, after creating a high-dimensional vector representation of each peptide in the library, the software package constructs an index structure that efficiently retrieves the closest library entries to a query spectrum. This approach is similar to Spectroscape. But there are several key differences in their default settings. First, ANN-Solo search for peptide candidates with a mass tolerance of 300Da or 500Da, while Spectroscape search as not mass tolerance constraint. Second, Spectroscape calculates a simple dot product, while ANN-Solo uses a more sophisticated similarity measure that considers mass-shifted peak matches. Third, ANN-Solo supports target-decoy search strategy to control FDR, while Spectroscape does not currently support FDR control.

To compare the speed of the two software tools, we used them to search two files in the PXD000561 dataset against the NIST Human HCD library (build May 19, 2020). The default search parameters of ANN-Solo were altered, with the maximum accepted m/z being 6000 and setting the "peak shifts" option to false, to better mimic Spectroscape's similarity measure. We found that while the index time of Spectroscape is about six times longer than that of ANN-Solo, Spectroscape search time is about three times faster (**Supplementary Table 1**). Since the indexing of a spectral library only needs to be done once for multiple searches, the search time comparison is more relevant practically.

Next, we analyzed the spectrum-spectrum matches (SSMs) filtered at 0.1% or 1% FDR by ANN-Solo, and compared them to the SSMs found by Spectroscape of the same spectra. We found that the two sets of SSMs had good agreement, with an overlap of around 70%. Furthermore, we observed that this overlap could be increased by 5% by counting the lower hits of Spectroscape

(up to rank 10), and by 2% if we consider sequences differing by one amino acid as matched (**Supplementary Table 2**).

Supplementary Note 2. Offline clustering and correction of unidentified and misidentified spectra

Although Spectroscape is primarily designed for on-demand visualization, its backend can potentially be used for offline spectrum clustering of the entire spectral archive. For any given query, Spectroscape returns a list of ANNs with their true dot products to the query. For each query spectrum, we have their list of ANNs and their true dot products. If we iterate over the whole archive, using each spectrum as a query to Spectroscape, we can construct an adjacency matrix from the returned list of ANNs. Using the adjacency matrix, we connect any pair of spectra with a true dot product greater than 0.7. Connected (directly or indirectly) spectra are considered one cluster. For illustrative purpose, we only connect spectra with the same precursor charge and similar precursor m/z (within 1 Th), such that spectra in the same cluster can be presumed to come from the same peptide ion. We applied a simple and relatively conservative heuristic to re-annotate unidentified and misidentified spectra: if 60% or more of the spectra in a cluster are annotated to the same peptide ion (by MSFragger at 1% FDR), we consider them the "majority" with the correct identification of the cluster and re-annotate all other spectra in the cluster to the same peptide ion. Of the over 15 million unidentified spectra in the PXD000561 dataset, about 4 million belong to a cluster that includes at least one identified member. (The rest are either not clustered with any other spectrum or belonging to a cluster with no identified member at all, and thus can never be re-annotated by clustering.) Out of those that can be potentially "rescued," 589,162 (14.7%) can be re-annotated by the 60% majority rule. Among them, 308,949 (52.4%) were actually identified by MSFragger to the same peptide ion, but fell short of the confidence threshold for 1% FDR, which partly validated this strategy of rescuing unidentified spectra. With the same heuristic, another 110,374 spectra with a conflicting identification with the majority are deemed misidentified and are re-annotated. In total, about nearly 700,000 spectra are re-annotated. The nodes of the re-annotated spectra are re-colored and displayed in Spectroscape with a 'R' in the middle of the node.

Supplementary Methods

Supplementary Method 1. The FAISS IVF-PQ algorithm

The Facebook AI Similarity Search (FAISS) library, published in 2019, has implemented a number of indexing algorithms that enable fast retrieval of similar digital objects (e.g. images, texts) among a huge pool of candidates. We adapted one of them, named inverted file index product quantization (IVF-PQ) for tandem mass spectra. The first step, called the "inverted file index" step, is to divide a high-dimensional space where a vector representation of a spectrum resides into regions (called "buckets"). This is done in a "training" step by k-means clustering of a randomly selected sample of 100,000 spectra. In brief, the algorithm searches for the partition of the data points into k buckets $\{C_1, C_2, C_3, \dots, C_k\}$ with corresponding centroids $\{c_1, c_2, c_3, \dots, c_k\}$ that minimizes the function:

$$D = \sum_{j=1}^{k} \sum_{x \in C_j} \left\| x - c_j \right\|^2$$

In the variant that we used in this study (short-handed "IVF256,PQ16"), 256 buckets were used. Next, for each vector x representing a spectrum, its nearest bucket centroid is found:

$$c(x) = \underset{c_j \in \{c_1, \dots, c_{256}\}}{\arg\min} \left\| x - c_j \right\|$$

Here $c(.)$ is the function that maps a vector to its nearest centroid. The residual vector is computed:

$$r = x - c(x)$$

Then the spaces of the residual vectors are divided into 16 subspaces in the "product quantization" step, such that r is represented by a *direct sum* of 16 256-dimensional vectors:

$$r = r^1 \oplus r^2 \oplus r^3 \oplus \cdots \oplus r^{16}$$

In our case, the decomposition into subspaces is achieved by randomly partitioning the 4096 bins along the m/z axis of the spectrum into 16 sets of 256 bins. Next, the 256-dimensional vectors in

each of the subspace are further sorted into 256 "sub-buckets" by k-means clustering, similar to the IVF step. Finally, the residual vector is approximated by a direct sum of the centroids of the sub-buckets, $\hat{r}$:

$$r \approx \hat{r} = c(r^1) \oplus c(r^2) \oplus c(r^3) \oplus \cdots \oplus c(r^{16})$$

The spectrum vector is finally approximated by:

$$x = c(x) + r \approx c(x) + \hat{r} = c(x) + [c(r^1) \oplus c(r^2) \oplus c(r^3) \oplus \cdots \oplus c(r^{16})]$$

The "address" of the spectrum vector is simply a concatenation of the bucket number and the 16 sub-bucket numbers. Both the bucket number and the sub-bucket number have 256 possible values, so each can be specified in 1 byte. Hence, the address is 17-byte long.

At query time, the query spectrum undergoes the same spectrum preprocessing steps, and its vector representation y is computed. The address is found by the same algorithm, as follows:

$$s = y - c(y)$$

$$y = c(y) + s \approx c(y) + \hat{s} = c(y) + [c(s^1) \oplus c(s^2) \oplus c(s^3) \oplus \cdots \oplus c(s^{16})]$$

where s is the residual vector of y, and $\hat{s}$ is the approximation of s based on the direct sum of centroids. To retrieve approximate nearest neighbors, the algorithm first finds the M nearest bucket centroids (M can be set by the query-time parameter *nprobes*), and collects all the existing addresses (each corresponding to a spectrum in the spectral archive) in those buckets. These are the candidates of the approximate nearest neighbors. However, instead of returning all these candidates, the algorithm performs efficient approximate dot product calculations between the query's address and the candidates' addresses:

$$\begin{aligned} x \cdot y &= (c(x) + r) \cdot (c(y) + s) = c(x) \cdot c(y) + c(x) \cdot s + r \cdot c(y) + r \cdot s \\ &\approx c(x) \cdot c(y) + c(x) \cdot \hat{s} + \hat{r} \cdot c(y) + \hat{r} \cdot \hat{s} \end{aligned}$$

Note that the dot product of two direct sums is distributive (provided the subspaces are the same), and can be written as a direct sum of dot products, e.g.:

$$\hat{r} \cdot \hat{s} = [c(r^1) \oplus c(r^2) \oplus c(r^3) \oplus \cdots \oplus c(r^{16})] \cdot [c(s^1) \oplus c(s^2) \oplus c(s^3) \oplus \cdots \oplus c(s^{16})]$$

$$= [c(r^1) \cdot c(s^1)] \oplus [c(r^2) \cdot c(s^2)] \oplus [c(r^3) \cdot c(s^3)] \oplus \cdots \oplus [c(r^{16}) \cdot c(s^{16})]$$

Thus, the approximate dot product is calculated by summing inter-centroid dot products. Since the centroids are known at training, all inter-centroid dot products can be computed beforehand and stored in a lookup table. Therefore, no multiplication is necessary and only about 50 additions are required for each approximate dot product calculation. This is further accelerated by parallel computing in GPUs. The algorithm then returns the most similar 1,024 approximate nearest neighbors (ANNs) among the candidates, as ranked by this approximate dot product.

To improve the recall, multiple distinct indices can be generated for the same spectral archive. To produce a different index, a different random sample of spectra is used in the training, and a different random partitioning of bins is used during the decomposition into subspaces in the product quantization step. At query time, each index will return its own set of 1,024 ANNs, many, but not all, of which will be common between indices. The union of the ANNs returned by all indices are passed to the next step for true dot product calculations and clustering.

In summary, the IVF-PQ algorithm approximates each spectrum vector as a sum of centroids for efficient dot product calculations. The address is 17-byte long, which means the addresses of 1 billion spectra can theoretically fit in 17 GB, small enough to be loaded into memory for most modern computers for fast processing. In terms of time complexity, retrieval of ANNs is expected to scale linearly with the archive size. This is because the number of spectra in each bucket, and hence the number of candidates for approximate dot product calculations, is expected to grow linearly with archive size. The efficient and parallelizable nature of the algorithm, however, helps to keep the running time manageable, especially with continuous hardware improvement (e.g., addition of more GPUs). Moreover, as more and more spectra are accumulated in spectral repositories, an increasing fraction of them (at least in their preprocessed and vectorized forms) will be identical or nearly identical, as they should ultimately originate from a finite set of observable peptide ions. Future development of the algorithm should aim to reduce this redundancy.

Supplementary Method 2. Optimization of the FAISS IVF-PQ algorithm for spectral archives

There are two hyper-parameters in the IVF-PQ algorithm to be optimized, *nprobes* and *nindices*. The former controls the number of nearby buckets to look into for each query, and the latter is the number of indices used in the spectral archive. Increasing *nprobes* and *nindices* will lead to higher recall, at the expense of computational time. We tested the retrieval performance of Spectroscape on settings of *nindices* ranging from 1 to 6, and *nprobes* ranging from 1 to 64 (**Supplementary Figure 1**). The overall recall of 10,000 randomly chosen queries was used as the metric. Although perfect per query recall (100%) is possible for higher values of *nprobes* and *nindices*, there is a diminishing improvement in recall as one increases *nindices* beyond 2 and *nprobes* beyond 8. Therefore the optimal hyper-parameter setting was chosen to be *nprobes* = 8 and *nindices* = 2, resulting in an overall recall over 98%. It should also be noted that perfect recall is not necessary for our application, as in a vast majority of cases, the identity of a query spectrum can be clearly determined provided we have a substantial fraction TNNs retrieved, since most spectra occur repeatedly in many replicates. Losing a few of them in the retrieval process will not materially affect the final outcome.

Supplementary Method 3. Comparison with GLEAMS in terms of ANN retrieval

Both GLEAMS and Spectroscape perform fast approximate nearest neighbor (ANN) retrieval by generating a low-dimensional approximation of each input spectrum. In GLEAMS, this is a 30-dimension vector embedding, and in Spectroscape, this is a 17-byte address, each byte corresponding to a centroid either in the global space or one of the 16 subspaces. For GLEAMs, each spectrum is represented as 32 floating point numbers, occupying 128 bytes in single precision. Spectroscope's address is much smaller.

Although both tools rely critically on dimensionality reduction to generate a compact representation of a spectrum, GLEAMs and Spectroscape are quite different functionally. GLEAMS is a clustering tool. It takes a list of data files and outputs a mapping from each individual spectrum to the spectrum cluster it belongs to. The embedding is trained with a deep neural network model to preserve the similarity of two spectra of the same peptide. In other words, in choosing the embedding deliberately, GLEAMS maximizes the chance that spectra of the same peptide will be placed in the same spectrum cluster, without directly computing spectral

similarities. It has no query of visualization function. Spectroscape, on the other hand, is a visualization tool of a spectral archive. The only input to Spectroscape's indexing algorithm are the peak lists of all the spectra, and Spectroscape is agnostic about the identification or additional information such as the precursor mass. The IVF-PQ indexing of Spectroscape seeks to approximate the spectrum geometrically in high-dimensional space, so that spectral similarities can be computed quickly for ANN retrieval. Giving a query spectrum, Spectroscape first uses the IVF-PQ index to retrieve its ANNs. Then it computes pairwise true spectral similarities among the ANNs and displays the neighborhood of any given query as a "network," or in computer science term, a graph. This is done in real time. Spectroscape does not cluster all the input spectra beforehand. Unlike GLEAMS, Spectroscape does not output the mapping of each individual spectrum to a spectrum cluster it belongs to. Rather, Spectroscape allows the user to verify any PSM by observing its "neighborhood" in a spectral archive. To facilitate this application, the identification results of all spectra are loaded and used to color-code the nodes of the graph, *after the fact*. It is worth emphasizing that unlike GLEAMS, Spectroscape does not take advantage of the information of the peptide identification or precursor mass in IVF-PQ indexing the ANN retrieval.

With this functional distinction of the two tools in mind, while we cannot compare the outputs of the two tools, we can compare their ability to preserve the true dot product of pairs of spectra in their respective spectrum representations ("embedding" for GLEAMS, "address" for Spectroscape). For any pair of spectra, we can calculate the true dot product, Spectroscape approximate dot product, and GLEAMS approximate dot product (of the corresponding embedding vectors). We searched 20,000 randomly selected queries against the spectral archive and collected the query-neighbor spectrum pairs and their Spectroscape approximate dot product. The true dot product scores between these spectrum pairs are also calculated. Note that Spectroscape returns about 2,000 query-neighbor spectrum pairs for each query, which would have filtered away pairs with very low similarity and of no practical relevance. The retained spectrum pairs thus form a more appropriate test set to evaluate the ability of the ANN retrieval algorithms. The corresponding GLEAMS approximate dot product scores between these same spectrum pairs are also calculated. (To correctly find the corresponding embedding of a spectrum, we use the filename and scan number as universal identifier for every spectrum.) We use scatter plots to visualize the correlation between the three scores, GLEAMS dot product vs true dot product,

Spectroscape dot product vs true dot product, and GLEAMS dot product vs Spectroscape dot product (**Figure 4a** of the main text).

Another key difference between Spectroscape and GLEAMS is that Spectroscape is effectively performing an “open” search without any precursor mass restriction, while GLEAMS embedding is trained with the information of peptide identification (if any) and the precursor mass. Therefore, we conjecture that when a pair of spectra are of the same precursor mass, GLEAMS embedding should be better able to preserve the true dot product, and will perform worse when the spectrum pair are of dissimilar precursor masses. To test this conjecture, in **Figure 4b and 4c** of the main text, we provide separate scatter plots for spectrum pairs for which the precursor masses are similar (within 3 Da) or dissimilar (at least 3 Da apart).

The speed of GLEAMS and Spectroscape are compared on an embedding or indexing task of 25 million spectra of the Human Proteome Project dataset (PXD000561). GLEAMS uses eight NVIDIA GeForce RTX 2080 Ti GPUs and Spectroscape the same type of GPU but only use one of them. GLEAMS takes 182 min to create for one embedding for each of the 25 million spectra and Spectroscape takes 156 min to create two indices of the 25 million spectra. The GLEAMS embedding takes 2.8 GB space, while Spectroscape indices only takes 1.2GB. Overall, Spectroscape is faster and more efficient in storage.

Supplementary Method 4. True dot product calculations

As described in the main text, the query spectrum is represented as 50 mz values, sorted by intensity in decreasing order and stored in the MZ file:

$$q = \{mz_0, mz_1, \dots, mz_{49}\}, mz_i \in \{0,1,\dots,65535\}$$

Here, 65535 is the largest integer that can be stored in 2 bytes, so we took maximum advantage of it to store the real m/z values $mz_{i,real}$ as accurately as possible:

$$mz_i = \left\lfloor \frac{mz_{i,real}}{2000} \times 65535 \right\rfloor$$

where $\lfloor\cdot\rfloor$ is the floor function. (We consider 2000 to be the maximum m/z value of a typical peptide tandem mass spectrum, and the rare m/z values beyond 2000 will be recorded as 65535.)

This enables us to maintain higher m/z compared to the 4096-dimension vector used for index building. Thus, the spectrum q is vectorized by binning the m/z axis into 65536 bins (with bin width 2000/65536 = 0.0305 m/z), resulting in a 65536-dimension vector $V(q)$:

$$V(q) = (v_0, v_2, v_3, v_4, \dots, v_{65535})$$

$$v_k = \begin{cases} 50 - i & if\ k = mz_i \\ 0 & otherwise \end{cases}$$

For calculating the accurate dot product, the query spectrum and the ANNs are retrieved from the MZ files. Then only the query spectrum is vectorized as described above. To mimic a wider m/z tolerance than the bin width, the same rank-transformed intensity ($50 - i$) is placed into neighboring bins for the query spectrum. For example, by default, a fragment ion m/z tolerance of approximately ± 0.045 Da is used for HCD spectra in Spectroscape, which is accomplished by filling the bins between $mz_i - 1$ and $mz_i + 1$ with the same rank-transformed intensity for each occupied bin at mz_i. Finally, the dot product between $V(q)$ and one of its ANNs, s, is calculated as:

$$dp(s, q) = \sum_{mz_i \in s} (50 - i) v_{mz_i}, v_{mz_i} \in V(q)$$

This efficient dot product calculation avoids any comparison of m/z values, and the vectorization of the spectra of the ANNs. The only steps required is to generate $V(q)$ from the query spectrum q, and the dot product calculation which requires 50 multiplications and 49 additions.

For determining the whole cluster structures among the retained TNNs, pairwise accurate dot product calculations are necessary. To do so, each of the TNNs take the place of the query spectrum q in the above algorithm.

Supplementary Method 5 | Visualization and force-directed graph drawing

Spectroscape displays the queried spectrum as a node in a graph, together with the top N true nearest neighbors (where N is 20 by default but can be adjusted at query time) in its "neighborhood." The true nearest neighbors (TNNs) were obtained by calculating accurate dot products between the query and the retrieved ANNs. For the displayed nodes of TNNs, pairwise

dot product calculations were performed to determine if they should be joined by an edge. The length of the edge is initialized to a function of the Euclidean distances between the vectorized spectra, as computed by:

$$d = \sqrt{\boldsymbol{x}^2 + \boldsymbol{y}^2 - 2\boldsymbol{x} \cdot \boldsymbol{y}}$$

If we assume vector $\boldsymbol{x}, \boldsymbol{y}$ are normalized, that is $\boldsymbol{x}^2 = \boldsymbol{y}^2 = 1$, then

$$d = \sqrt{1 + 1 - 2\boldsymbol{x} \cdot \boldsymbol{y}} = \sqrt{2(1 - \boldsymbol{x} \cdot \boldsymbol{y})}$$

where $\boldsymbol{x} \cdot \boldsymbol{y}$ is the dot product, and $0 < d < \sqrt{2}$. Only edges corresponding to distances below a certain threshold, which defaults to 0.8 but can be user-specified.

However, instead of keeping the edge lengths as the calculated Euclidean distances between the spectra, the graph of nodes (representing spectra) is plotted using a force-directed graph algorithm, implemented in D3.js (https://d3js.org/), which is a free JavaScript library focused on interactive data visualization. The idea is to run a physical simulation in which nodes are spheres of mass $m = 1$ unit, and charge $c = 1$ unit and edges are massless springs with desired link distance l and spring constant $k = 1$ unit. The elastic force between two joined nodes can be calculated by Hooke's law:

$$F = -k\Delta l$$

and the repulsion between two charged spheres can be calculated by Coulomb's law.

$$F = -k_e \frac{q_1 q_2}{l^2}$$

In addition, a gravity force pointing to the center of the drawing canvas is included to balance the repulsive force. The node corresponding to the query spectrum is fixed at the center and displayed as a larger circle than the neighbors. Starting from any initial state, the whole system will evolve with Newton's law of motion, where the acceleration is used to update the velocity, and velocity is used to update the position of the node. This simulation will reach an equilibrium where every node's net force added up to zero. After some testing to optimize for simulation speed, the length

of the edge (the massless spring) is initialized to be a logistic transformation of the Euclidean distance:

$$l = 30 + \frac{80}{1 + e^{20d-12}}$$

Force-directed graph drawing can minimize crossing edges and overlapping nodes in graph drawing. More importantly, it has the effect of clearly displaying clusters of densely-connected nodes, while sending loosely-connected nodes farther away, facilitating the detection of sub-clusters, outliers and bridges.

The user can also choose to disconnect the edges between neighbors regardless of their similarities, so the resulting graph is star-shaped with the query spectrum in the middle. The cluster structure will not come into play. Concentric circles marking a scale of Euclidean distances from the center (query) node can be overlaid on the graph for easy visualization (**Supplementary Figure 4**).

Mousing over any node will display information about the node, including its identification (if any), precursor m/z, confidence of identification (shown as PeptideProphet and iProphet probabilities), source file and scan number, etc. Single-clicking any node will display its corresponding full spectrum in a spectrum viewer, with peak annotation if the identification is known. The user can also further explore the displayed "neighborhood" by double clicking on any node, which will initiate a new query with the clicked node as the query spectrum, replacing the old one. Alternatively (in an option available in a menu opened by right-clicking), one can expand the neighborhood by adding neighbors of any clicked node, without replacing the old graph.

An option also exists for the user to enter a peak list into the web interface in a text box (in which each line is a peak represented by the m/z and the intensity, separated by space), and search it against the archive. This query spectrum is not added into the archive, but Spectroscape will retrieve its nearest neighbors among all spectra in the archive and display them as nodes in clusters (**Supplementary Figure 5**).
