# Supplementary material for: Spectroscape enables real-time query and visualization of a spectral archive in proteomics
Source: Nat Commun. 2023 Oct 7;14:6267. doi: 10.1038/s41467-023-42006-x (PMC10560257; doi:10.1038/s41467-023-42006-x)
Supplement: Supplementary file 3 — Reporting Summary [file 41467_2023_42006_MOESM3_ESM.pdf]

nature portfolio

Corresponding author(s): Henry Lam

Last updated by author(s): Sep 14, 2023

Reporting Summary

Nature Portfolio wishes to improve the reproducibility of the work that we publish. This form provides structure for consistency and transparency in reporting. For further information on Nature Portfolio policies, see our Editorial Policies and the Editorial Policy Checklist.

Statistics

For all statistical analyses, confirm that the following items are present in the figure legend, table legend, main text, or Methods section.

n/a	Confirmed	
☒	☐	The exact sample size (*n*) for each experimental group/condition, given as a discrete number and unit of measurement
☐	☒	A statement on whether measurements were taken from distinct samples or whether the same sample was measured repeatedly
☒	☐	The statistical test(s) used AND whether they are one- or two-sided<br>*Only common tests should be described solely by name; describe more complex techniques in the Methods section.*
☒	☐	A description of all covariates tested
☒	☐	A description of any assumptions or corrections, such as tests of normality and adjustment for multiple comparisons
☒	☐	A full description of the statistical parameters including central tendency (e.g. means) or other basic estimates (e.g. regression coefficient) AND variation (e.g. standard deviation) or associated estimates of uncertainty (e.g. confidence intervals)
☒	☐	For null hypothesis testing, the test statistic (e.g. *F*, *t*, *r*) with confidence intervals, effect sizes, degrees of freedom and *P* value noted<br>*Give P values as exact values whenever suitable.*
☒	☐	For Bayesian analysis, information on the choice of priors and Markov chain Monte Carlo settings
☒	☐	For hierarchical and complex designs, identification of the appropriate level for tests and full reporting of outcomes
☒	☐	Estimates of effect sizes (e.g. Cohen's *d*, Pearson's *r*), indicating how they were calculated

Our web collection on statistics for biologists contains articles on many of the points above.

Software and code

Policy information about availability of computer code

Data collection	n/a. No software was used for data collection.
Data analysis	Spectroscape is implemented in C++ and JavaScript and is freely available as open-source software under MIT license at https://github.com/wulongict/SpectralArchive/ for individual groups to build their own spectral archive. Detailed installation and running instructions are provided in the README file. The following third party open source code packages were included in the Spectroscape distribution: FAISS v1.7.3 (used for building indices to retrieve approximate nearest neighbors), Boost v1.65.1, gtest v1.7.0, rapidxml v1.13, spdlog v1.x, eigen v3.3.1, MSToolkit (disseminated with cometv2016, used to read the mzXML and mzML formats), and SpectraST v5.0 (used to read the splib format).<br>MSConvert in ProteoWizard v3.0.19038 is used to convert raw to mzXML format.<br>MSFragger v20190628 is used to search mzXML files against human sequence database.<br>PeptideProphet and iProphet in Trans Proteomic Pipeline (TPP) v6.0.0 were used to processed the search results.<br>Nginx v1.18.0 and spawn-fcgi v1.6.4 are used for web service of Spectroscape.<br>D3.js v4.0, jQuery v3.3.1 and bootstrap v4.1.1 are in the web interface.

For manuscripts utilizing custom algorithms or software that are central to the research but not yet described in published literature, software must be made available to editors and reviewers. We strongly encourage code deposition in a community repository (e.g. GitHub). See the Nature Portfolio guidelines for submitting code & software for further information.

Data

Policy information about availability of data

All manuscripts must include a data availability statement. This statement should provide the following information, where applicable:

- Accession codes, unique identifiers, or web links for publicly available datasets
- A description of any restrictions on data availability
- For clinical datasets or third party data, please ensure that the statement adheres to our policy

The data used for building the spectral archive was downloaded from ProteomeXchange data repository with identifer PXD000561 and PXD010154. These two data sets are described in Kim et al., "A Draft Map of the Human Proteome." Nature 509, 575-581 (2014) and Wang et al., "A deep proteome and transcriptome abundance atlas of 29 healthy human tissues." Mol Syst Biol 15, e8503 (2019), respectively.
The human sequence database was downloaded from UniProt with accession id UP000005640 (dated 2021-09-06).
The human NIST libraries were downloaded from https://chemdata.nist.gov/dokuwiki/doku.php?id=peptidew:lib:humanhcd20160503 (dated 2020-05-19).

Research involving human participants, their data, or biological material

Policy information about studies with human participants or human data. See also policy information about sex, gender (identity/presentation), and sexual orientation and race, ethnicity and racism.

Reporting on sex and gender	n/a
Reporting on race, ethnicity, or other socially relevant groupings	n/a
Population characteristics	n/a
Recruitment	n/a
Ethics oversight	n/a

Note that full information on the approval of the study protocol must also be provided in the manuscript.

Field-specific reporting

Please select the one below that is the best fit for your research. If you are not sure, read the appropriate sections before making your selection.

☒ Life sciences ☐ Behavioural & social sciences ☐ Ecological, evolutionary & environmental sciences

For a reference copy of the document with all sections, see nature.com/documents/nr-reporting-summary-flat.pdf

Life sciences study design

All studies must disclose on these points even when the disclosure is negative.

Sample size	We built an spectral archive with over 100 million MS2 spectra. This is the same magnitude as the total spectra of MASSIVE-KB as is mentioned in Bittremieux, W., May, D.H., Bilmes, J. et al. A learned embedding for efficient joint analysis of millions of mass spectra. Nat Methods 19, 675–678 (2022). The sample size was chosen to be as large as data availability allows, to demostrate the scalability of the software tool.
Data exclusions	None
Replication	To illustrate the efficiency of Spectroscape, six IVF-PQ indices are built using random samples and different partition of the m/z bins. The search speed of Spectroscape is shown as the average of the six indices.
Randomization	The six indices of spectral archives with 100k spectra randomly selected from the 25 million spectra. This step is unsupervised learning, like k-means algorithm, therefore, there is no need to split training and testing set.<br>The recall of Spectroscape is evaluated on spectral archive with different number of MS2 spectra, ranging from 1 million to 100 million by searching 20,000 randomly selected queries spectra from the spectral archive. And the recall is averaged over the 20,000 random queries.
Blinding	To evaluate the overall recall by IVF-PQ indices, we randomly selected query spectra as test set, and calculated their true nearest neighbors (TNNs) by brute-force search of the whole archive. This makes investigator blind to test set, and the ground truth.

Reporting for specific materials, systems and methods

We require information from authors about some types of materials, experimental systems and methods used in many studies. Here, indicate whether each material, system or method listed is relevant to your study. If you are not sure if a list item applies to your research, read the appropriate section before selecting a response.

Materials & experimental systems

n/a	Involved in the study
☒	☐ Antibodies
☒	☐ Eukaryotic cell lines
☒	☐ Palaeontology and archaeology
☒	☐ Animals and other organisms
☒	☐ Clinical data
☒	☐ Dual use research of concern
☒	☐ Plants

Methods

n/a	Involved in the study
☒	☐ ChIP-seq
☒	☐ Flow cytometry
☒	☐ MRI-based neuroimaging
